# Increasing the use of second-line therapy is a cost-effective approach to prevent the spread of drug-resistant HIV: a mathematical modelling study

**DOI:** 10.7448/IAS.17.1.19164

**Published:** 2014-12-05

**Authors:** Brooke E Nichols, Kim CE Sigaloff, Cissy Kityo, Raph L Hamers, Rob Baltussen, Silvia Bertagnolio, Michael R Jordan, Timothy B Hallett, Charles AB Boucher, Tobias F Rinke de Wit, David AMC van de Vijver

**Affiliations:** 1 Department of Viroscience, Erasmus Medical Center, University Medical Center, Rotterdam, The Netherlands; 2 Department of Global Health, Academic Medical Centre, Amsterdam Institute for Global Health and Development (AIGHD), University of Amsterdam, Amsterdam, The Netherlands; 3 Joint Clinical Research Centre (JCRC), Kampala, Uganda; 4 Department of Primary and Community Care, Radboud University Nijmegen Medical Center, Nijmegen, The Netherlands; 5 HIV Department, World Health Organization, Geneva, Switzerland; 6 Department of Geographic Medicine and Infectious Disease, Tufts University School of Medicine, Boston, MA, USA; 7 Department of Infectious Disease Epidemiology, Imperial College London, London, UK; 8 PharmAccess Foundation, Amsterdam, The Netherlands

**Keywords:** drug resistance, second-line treatment, pre-therapy genotyping, viral load monitoring, cost-effectiveness, antiretroviral therapy

## Abstract

**Introduction:**

Earlier antiretroviral therapy (ART) initiation reduces HIV-1 incidence. This benefit may be offset by increased transmitted drug resistance (TDR), which could limit future HIV treatment options. We analyze the epidemiological impact and cost-effectiveness of strategies to reduce TDR.

**Methods:**

We develop a deterministic mathematical model representing Kampala, Uganda, to predict the prevalence of TDR over a 10-year period. We then compare the impact on TDR and cost-effectiveness of: (1) introduction of pre-therapy genotyping; (2) doubling use of second-line treatment to 80% (50–90%) of patients with confirmed virological failure on first-line ART; and (3) increasing viral load monitoring from yearly to twice yearly. An intervention can be considered cost-effective if it costs less than three times the gross domestic product per capita per quality adjusted life year (QALY) gained, or less than $3420 in Uganda.

**Results:**

The prevalence of TDR is predicted to rise from 6.7% (interquartile range [IQR] 6.2–7.2%) in 2014, to 6.8% (IQR 6.1–7.6%), 10.0% (IQR 8.9–11.5%) and 11.1% (IQR 9.7–13.0%) in 2024 if treatment is initiated at a CD4 <350, <500, or immediately, respectively. The absolute number of TDR cases is predicted to decrease 4.4–8.1% when treating earlier compared to treating at CD4 <350 due to the preventative effects of earlier treatment. Most cases of TDR can be averted by increasing second-line treatment (additional 7.1–10.2% reduction), followed by increased viral load monitoring (<2.7%) and pre-therapy genotyping (<1.0%). Only increasing second-line treatment is cost-effective, ranging from $1612 to $2234 (IQR $450-dominated) per QALY gained.

**Conclusions:**

While earlier treatment initiation will result in a predicted increase in the proportion of patients infected with drug-resistant HIV, the absolute numbers of patients infected with drug-resistant HIV is predicted to decrease. Increasing use of second-line treatment to all patients with confirmed failure on first-line therapy is a cost-effective approach to reduce TDR. Improving access to second-line ART is therefore a major priority.

## Introduction

In 2012, an estimated 2.4 million people became newly infected with HIV-1 globally [1]. Alongside behaviour change, male circumcision and condom use, the need for additional HIV prevention strategies remains. The initiation of antiretroviral therapy (ART) at a CD4 cell count between 350 and 550 cells/µl has the potential to prevent 96% of new infections as compared to treatment initiation at CD4 <250 cells/µl among sero-discordant couples [2,3]. In addition, a 41% reduction in mortality and opportunistic infections has been observed in individuals initiating ART at higher CD4 cell counts [2]. The World Health Organization (WHO) has recently revised its treatment guidelines and now recommends treatment initiation at CD4 <500 cells/µl [3,4].

There is concern that earlier ART initiation (i.e. at higher CD4 cell counts) may result in increased emergence and subsequent transmission of drug-resistant HIV [5]. This could in turn jeopardize the effectiveness of future HIV treatment, particularly in the context of restricted drug availability in many resource-limited countries. In a previous study, we predicted that as more individuals initiate ART early, far more new infections are averted than drug-resistant infections are gained [5]. Despite the predicted reduction in new drug-resistant infections, strategies to minimize drug resistance will remain essential to preserve the effectiveness of currently available drugs.

There are several ART programme-level strategies that can help mitigate the emergence and transmission of drug resistance [5–7]. WHO has recently recommended monitoring patients by measuring plasma HIV RNA level, or viral load testing, which can reduce transmitted drug resistance (TDR) if implemented at regular intervals (every 6 or 12 monthly). viral load testing can reduce the emergence of HIV drug resistance by early identification of patients with virological failure, prompting intensified adherence counselling and switch to second-line ART as necessary, thereby minimizing emergence of HIV drug resistance [6,7]. Second, prompt switching to a protease-inhibitor (PI)-based second-line regimen of individuals experiencing virological failure has been associated with a reduced risk for drug resistance [5,8]. Finally, pre-therapy genotypic resistance testing to select a fully active regimens guide may mitigate acquired drug resistance [9,10]. However, these three strategies carry additional costs and are not routinely available in sub-Saharan Africa.

Mathematical modelling in combination with cost-effectiveness analyses can be used to help inform policy makers about ways to prevent new HIV infections while simultaneously minimizing TDR, at the lowest possible cost. The aim of this analysis was to determine the most cost-effective of strategy that can be used to prevent the spread of TDR in settings with similar characteristics of Kampala, Uganda.

## Methods

### Study design and population

We used a previously published compartmental deterministic mathematical model [5] based on an urban population in Kampala. To predict time trends of TDR our model included drug resistance data from the PharmAccess African Studies to Evaluate Resistance (PASER) on transmitted [11] and acquired [9,12] drug resistance in Kampala.

### Model and calibration

The model has been extended to incorporate population growth of the catchment area of the Joint Clinical Research Centre (JCRC), further expansion of ART and different patient monitoring strategies that can be used to reduce drug resistance. Using Monte Carlo filtering techniques [13], we accepted 1438 of 515,000 simulations that were associated with a specified TDR prevalence [14], proportion of mutations observed in TDR, HIV prevalence and population size (Supplementary Table 1 shows the values used for calibration). The model calibration to the population size and HIV prevalence is shown in the supplement (Supplementary Figures 1 and 2). All reported results are the median and interquartile range (IQR) of the accepted simulations.

At the JCRC, the HIV test rate is relatively low, as approximately 50% of individuals are tested with and initiate ART at CD4 counts <200 cells/µl. Therefore, even if immediate treatment was recommended upon diagnosis, we would expect no more than 10% of individuals to initiate at a CD4 threshold of >500 cells/µl (Supplementary Figure 3 shows this proportion of treatment initiation over time) assuming no change in the rate of HIV testing. Yearly viral load measurements and twice yearly CD4 cell counts are obtained for all patients on ART. After a detectable viral load, adherence counselling is provided, and thereafter a second viral load measurement is obtained. Pre-therapy genotypic testing is not provided.

In accordance with PASER-Monitoring data, the proportion of people who switch to second-line therapy with confirmed virological failure (defined as a plasma HIV RNA value of ≥1000 copies/mL) after adherence counselling during the first two years on therapy is 33–66% of those on tenofovir-based regimens and 33–50% of those on zidovudine-based regimens [5]. This resulted in approximately 3.5% of patients switching to second-line therapy after one year [5]. Of the individuals with virological failure during the second year of antiretroviral treatment, a median of 33% (range 16–48%) had viral resuppression on their tenofovir-based regimens and a median of 10% (range 8–21%) on zidovudine-based regimens [5]. We assumed that these percentages of switching to second-line and resuppression on first-line would persist beyond two years on therapy. This would result in many individuals failing on first-line therapy to be switched to second-line over several years. Higher rates of switching to second-line would result in individuals switching earlier after initial virological failure, on average. The switch rate at the JCRC is not CD4 cell count-dependent. Approximately 40% of individuals receive tenofovir-containing regimens at the JCRC, and 60% zidovudine-containing regimens, both combined with emtricitabine or lamivudine and efavirenz or nevirapine [5]. Table 1 shows the key assumptions for this model.

**Table 1:** Key model parameters [5]

Description	Estimate or range^a^	Reference
Disease stages duration		[15,16]
Acute stage	10–16 weeks	
Chronic stage >500 cells/µl	0.87–1 year	
Chronic stage 350–500 cells/µl	2.9–3.1 years	
Chronic stage 200–350 cells/µl	3.6–3.9 years	
AIDS stage^b^	6–12 months	
Final AIDS stage^b^	7–13 months	
Infectivity		[17,18]
Acute stage	27–43 times that of chronic stage	
Chronic stage (all)	10% per year	
AIDS stage^b^	3–5 times higher than chronic stage	
Final AIDS stage^b^	0%	
Proportion of people in sexual risk		Model calibration
Highest	1.5–2.5%	
Second	10–20%	
Third	20–30%	
Lowest	47.5–68.5%	
Number of partners per year in each sexual risk group		Model calibration
Highest	9–14	
Second	1.7–3	
Third	0.12–0.22	
Lowest	0.04–0.06	
Mortality rates per year		[19]
Population	0.02	
Chronic HIV stage	0.098	
AIDS stage	0.63	
On treatment during chronic stage, first year	0.02–0.098	
On treatment during chronic stage, 12+ months	0.02–0.05	
On treatment during AIDS stage, first year	0.03–0.3	
On treatment during AIDS stage, 12+ months	0.03–0.06	
HIV test rate		
Baseline	10–30%	Model calibration
Rate of being tested in the acute stage of HIV	Half of the test rate	Assumption^c^
Rate of being tested in the chronic stage of HIV	Test rate	Model calibration
Rate of being tested in the AIDS stage	Test rate+10%	
Linkage to care from test to treat	75–100%	Model calibration
Reduction in transmissibility of those	90–100%	[2,20,21]
Percentage of people that go to second-line after continued virological failure, yearly after 12 months on treatment:		
On zidovudine-based regimen	33–50%	PASER-monitoring, Kampala
On tenofovir-based regimen	33–66%	
Percentage of those who go onto second-line not due to resistance in the first 12 months	1.5–3%	[22]

a All ranges are uniformly distributed.

b Two AIDS stages were included because during the final months before death, patients have limited sexual activity.

c Due to window phase of antibody-based test.

### Baseline scenarios

Three baseline scenarios, treatment initiation at CD4 <350 cells/µl, CD4 <500 cells/µl and immediate treatment upon diagnosis, were considered in this analysis. In our baseline scenarios, we assume yearly viral load monitoring for patients on treatment. We assumed that these monitoring approaches and switching rates from first- to second-line described above would persist unchanged. The laboratory monitoring and/or the increase in the use of second-line were subsequently evaluated for each treatment initiation threshold.

### Strategies to reduce TDR

At each CD4 initiation threshold, we evaluated scenarios in which we altered three patient monitoring strategies in order to reduce TDR. All strategies were modelled to be implemented in 2014, scaled-up linearly until 2016, and implemented until 2024. The first strategy is increased viral load monitoring every six months (instead of the current practice of yearly viral load measurements). We also evaluated the scenario where the biannual viral load measurements are provided for just the first two years on treatment. In the scenarios that evaluate biannual viral load alone, there is no increased access to second-line treatment but the yearly rate of resuppression on first-line is doubled.

Second, we evaluated a scenario with increased switch rate to second-line treatment. In this scenario, individuals with virological failure on first-line therapy after a yearly viral load measurement and do not achieve viral resuppression on first-line ART after adherence counselling (median resuppression rate 17.7%; range 8.3–49.3%) are switched to a second-line regimen after a confirmatory viral load test. Those who do not achieve viral resuppression on first-line ART after adherence counselling are then switched to second-line therapy (median 82.3%; range 50.7–91.7%). This scenario was also combined with biannual viral load testing.

Third, a scenario was evaluated where pre-therapy genotyping is performed for all individuals. Based on the resistance profile, a fully-active first-line regimen then prescribed.

### Cost-effectiveness analysis

Each compartment in our deterministic model was assigned a range of cost and quality adjusted life year (QALY) depending on the intervention (Table 2 shows key costs, and Supplementary Tables 1–4 show detailed costs and QALY assumptions) [24]. Rates of HIV clinical monitoring tests were taken from the JCRC's standard practice (Supplementary Table 5). Local costs for hospitalization of HIV infected persons, opportunistic infections, HIV testing, and ART, were all taken into account. Generally, a health-related intervention can be considered very cost-effective at a cost less than the gross domestic product (GDP) per capita ($1140 in Uganda in 2012 [25]) per QALY, and cost-effective if less than three times the GDP per capita ($3420) per QALY gained [26,27]. We calculated both the average cost-effectiveness ratios (ACERs) where we compared each scenario to baseline, and the incremental cost-effectiveness ratios (ICERs) where we compared each scenario to the next least-costly scenario [28]. Patient monitoring strategies were compared within each respective treatment initiation threshold (CD4 <350 cells/µl, <500 cells/µl, and immediate treatment). All costs and QALYs have been discounted yearly at the standard of 3% [29,30].

**Table 2:** Key cost parameters^a^

Description	Estimate^b^
Cost of testing negative for HIV per test^c^	$6
Cost of testing positive for HIV per test^c^	$21
Cost of an outpatient visit in the hospital^d^	$16
Cost of first inpatient day in the hospital^d^	$24
Cost of subsequent inpatient day in the hospital^d^	$8
Cost of zidovudine-based treatment, per year	$108
Cost of tenofovir-based treatment, per year	$223
Cost of boosted protease inhibitor-based treatment, per year (second-line therapy)	$268
Cost of a CD4 cell count^e^	$30
Cost of a viral load test^e^	$71
Cost of pre-therapy genotypic testing^e^	$159
Exchange rate, Ugandan Shilling to USD over year 2012	2500:1

a All costs collected from the Joint Clinical Research Centre in Kampala, Uganda.

b All costs are log-normally distributed±10% of the listed cost [23].

c Includes costs of HIV tests, outpatient staff, laboratory personnel.

d Includes costs related to infrastructure, nurses, doctors and other hospital personnel.

e Includes the price of an outpatient visit, costs of respective test and laboratory personnel.

### Sensitivity analysis

We performed a univariate sensitivity analysis of cost-effectiveness of second-line at each treatment initiation threshold. Six key input variables – cost of viral load testing, cost of CD4 cell count testing, cost of antiretroviral drugs, prevalence of TDR, cost discounting and QALY discounting – were considered to identify the sensitivity of our model. To evaluate whether the costs of viral load monitoring or pre-therapy genotyping influenced the cost-effectiveness of the scenarios including those tests, we calculated the ICERs for those scenarios with a reduction in the price of each up to 90%.

Availability of second-line treatment is limited throughout sub-Saharan Africa. Access to second-line treatment at the JCRC is, however, high. Therefore, we also performed a sensitivity analysis in which we assumed that second-line treatment is only limitedly available, as might be more representative for other African sites. We modelled this limited availability by reducing the number of people switching to second-line by 50–70% (thus on average, 8.8% of all patients on second-line treatment at 10 years in the limited second-line scenario, compared to 22% in the full scale-up of second-line, when treating at CD4 <350 cells/µl). We then calculated the impact on levels of TDR as well as the cost-effectiveness of switching all individuals with confirmed virological failure on first-line therapy to second-line therapy.

## Results

### Impact of ART on TDR

**Figure 1:**
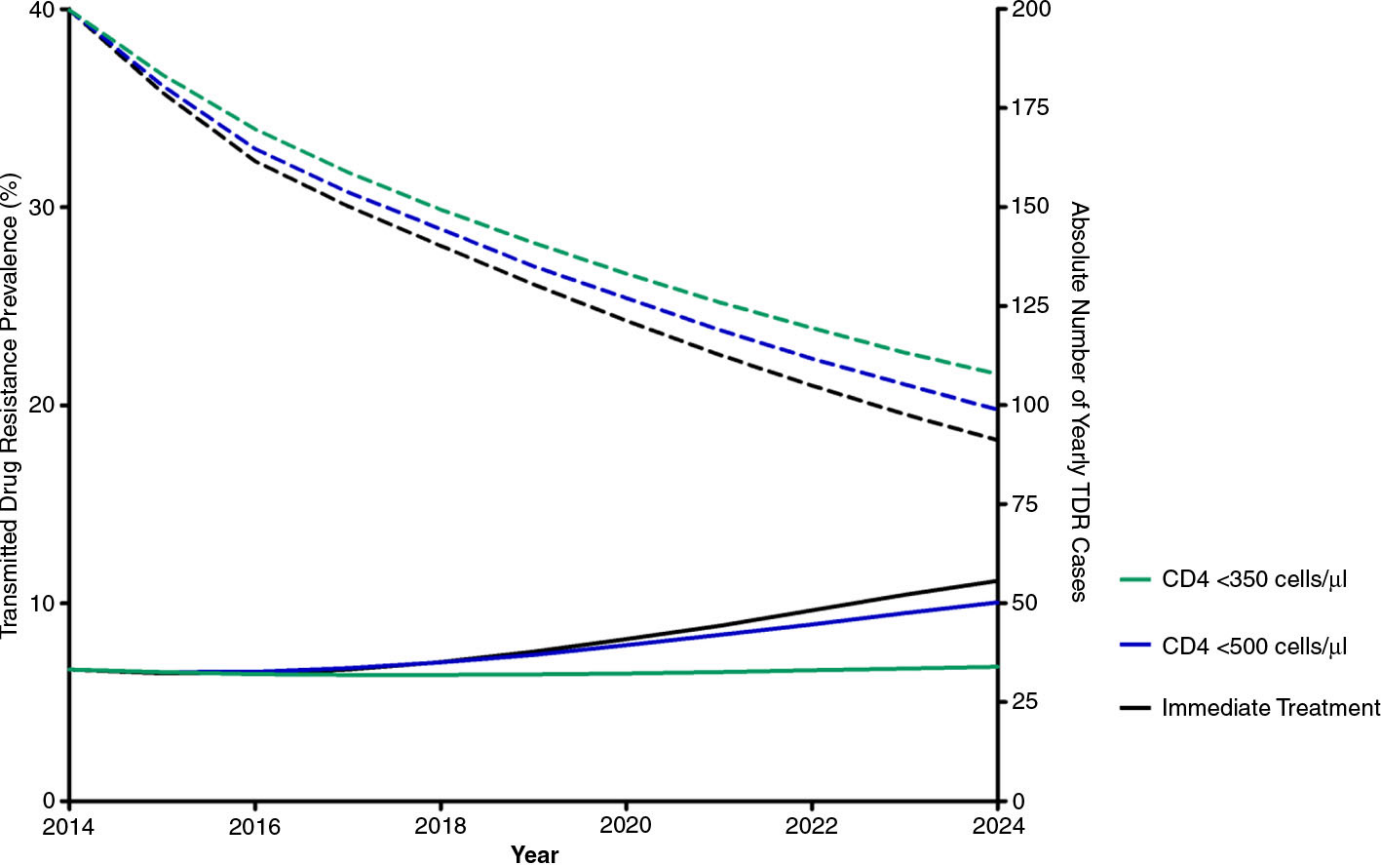
Yearly transmitted drug resistance prevalence (solid lines) and absolute number of yearly TDR cases (dashed lines) by CD4 treatment initiation thresholds of <350, <500 CD4 cells/µl, and immediate treatment over 10 years.

The prevalence of TDR is predicted to rise at all CD4 initiation thresholds (Figure 1). In 2014, the prevalence of TDR is predicted to rise from 6.7% (IQR 6.2–7.2%), to 6.8% (IQR 6.1–7.6%), 10.0% (IQR 8.9–11.5%) and 11.1% (IQR 9.7–13.0%) in 2024 if the treatment initiation threshold is CD4 cells <350 cells/µl, <500 cells/µl, and irrespective of CD4 cell count, respectively.

The absolute number of TDR infections is predicted to decrease, however, compared to initiating treatment at CD4 <350 cells/µl due to decreasing HIV incidence. Initiating treatment at a CD4 count of <500 cells/µl and treating immediately averts 61 or 4.4% (IQR 44–81 or 3.3–5.5%) and 110 or 8.1% (IQR 87–142 or 6.6–9.4%) of TDR infections, respectively, as compared to initiating ART at CD4 <350 cells/µl. TDR is predicted to be primarily due to NNRTIs, followed closely by resistance to PIs (Supplementary Figure 4).

### Epidemiological impact of strategies to reduce drug resistance

#### Biannual viral load monitoring

**Figure 2:**
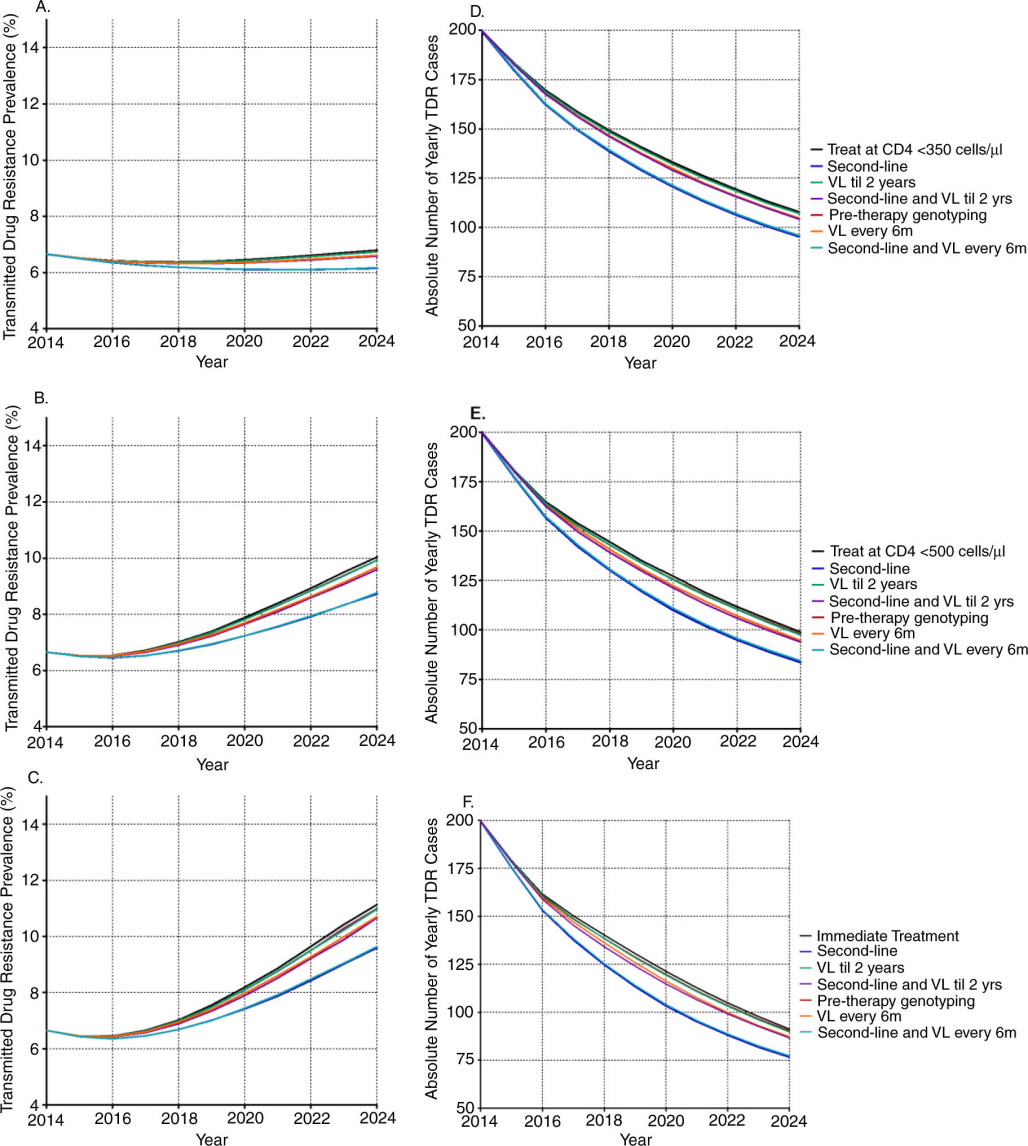
Yearly transmitted drug resistance prevalence (A–C) and absolute number (D–F) of yearly TDR cases by antiretroviral treatment initiation threshold, by patient monitoring strategy, over a period of 10 years. Panel A and D refer to when all monitoring strategies are implemented in combination with treatment initiation at CD4 <350 cells/µl, panel B and E in combination with treatment initiation at CD4 <500 cells/µl, and panel C and F in combination with immediate treatment. VL = viral load testing.

Biannual viral load monitoring had a modest impact on preventing new TDR infections (Figure 2). No more than 2.7% of TDR was predicted to be averted over 10 years at any treatment initiation threshold. The two viral load strategies (in which six-monthly viral loads were available for the first two years on therapy both with and without additional access to second-line) had minimal impact on TDR, averting <1.0% of TDR over the coming 10 years.

#### Increase in second-line

Increasing the use of second-line ART has the largest impact on averting drug-resistant infections (Figure 2). The largest effect of increased access to second-line was predicted when ART is initiated at time of diagnosis (averting 10.2% of TDR, IQR 8.5–12.0%), followed by treatment initiation at CD4 cell counts <500 cells/µl (9.4%, IQR 7.8–11.2%), and at <350 cells/µl (7.1%, IQR 5.8–8.5%), compared to the respective baseline scenarios at each treatment initiation threshold. Combining biannual viral load testing for the duration of ART with increased use of second-line ART did not greatly increase the impact on TDR compared to increasing use of second-line ART alone.

#### Pre-therapy genotyping

Pre-therapy genotyping had only a limited impact on preventing spread of drug-resistant HIV, averting a maximum of 1.0% of TDR over 10 years (Figure 2).

### Cost-effectiveness of strategies to reduce drug resistance

Increasing use of second-line treatment was the only strategy that was considered cost-effective in our analysis, with an ICER ranging between $1612 and $2234 per QALY gained depending on the treatment initiation threshold (Table 3). All other scenarios were dominated by increasing use of second-line, as all scenarios were more costly and less effective than second-line alone.

**Table 3:** Cost-effectiveness of strategies to reduce transmitted drug resistance by treatment initiation threshold

Intervention	Total cost (millions USD)	QALYs gained	Infections averted	Average cost-effectiveness ratio	Incremental cost- effectiveness ratio	Conclusions
**Treatment at CD4 <350**	33.8 (31.6–36.0)					
Increase second-line	34.0 (31.7–36.2)	81 (−199–351)	104 (83–130)	$1925 ($450-dominated)	$1925 ($450-dominated)	Cost-effective
Viral load every 6 months, for first 2 years on treatment	34.1 (31.8–36.4)	3 (−250–280)	9 (6–12)	$95,417 ($1077-dominated)	Dominated ($725-dominated)	Dominated
Viral load every 6 months, for first 2 years on treatment and increased second-line	34.2 (31.9–36.4)	29 (−234–287)	31 (23–40)	$11,602 ($1216-dominated)	Dominated ($908-dominated)	Dominated
Pre-therapy genotyping	34.6 (32.3–37.0)	3 (−285–273)	7 (5–10)	$329,018 ($3014-dominated)	Dominated ($3387-dominated)	Dominated
Continual viral load every 6 months	38.4 (35.9–41.0)	16 (−258–297)	25 (18–34)	$283,020 ($15,844-dominated)	Dominated ($24,765-dominated)	Dominated
Continual viral load every 6 months and increased second-line	38.5 (35.9–41.1)	75 (−170–329)	98 (75–126)	$64,539 ($13,884-dominated)	Dominated ($18,177-dominated)	Dominated
**Treatment at CD4 <500**	38.5 (36.0–41.3)					
Increase second-line	38.7 (36.2–41.5)	87 (−190–375)	132 (105–165)	$2234 ($505-dominated)	$2234 ($505-dominated)	Cost-effective
Viral load every 6 months, for first 2 years on treatment	38.9 (36.4–41.7)	−12 (−302–288)	12 (8–18)	Dominated ($1431-dominated)	Dominated	Dominated
Viral load every 6 months, for first 2 years on treatment and increased second-line	39.0 (36.5–41.8)	26 (−277–318)	43 (33–56)	$18,337 ($1473-dominated)	Dominated ($1290-dominated)	Dominated
Pre-therapy genotyping	39.6 (37.1–42.5)	−18 (−298–309)	13 (9–17)	Dominated ($3734-dominated)	Dominated	Dominated
Continual viral load every 6 months	44.0 (41.1–47.1)	2 (−290–310)	33 (24–43)	Dominated ($28,250-dominated)	Dominated	Dominated
Continual viral load every 6 months and increased second-line	44.0 (41.2–47.2)	70 (−208–366)	122 (95–157)	$72,975 ($15,593-dominated)	Dominated ($21,720-dominated)	Dominated
**Treat immediately**	39.9 (37.4–42.9)					
Increase second-line	40.1 (37.5–43.0)	121 (−165–406)	137 (109–169)	$1612 ($463-dominated)	$1612 ($463-dominated)	Cost-effective
Viral load every 6 months, for first 2 years on treatment	40.4 (37.7–43.4)	17 (−292–331)	13 (9–19)	$25,767 ($1276-dominated)	Dominated ($1315-dominated)	Dominated
Viral load every 6 months, for first 2 years on treatment and increased second-line	40.4 (37.8–43.4)	33 (−276–344)	46 (34–58)	$15,100 ($1517-dominated)	Dominated ($1544-dominated)	Dominated
Pre-therapy genotyping	41.2 (38.5–44.2)	18 (−329–341)	14 (10–19)	$69,252 ($3620-dominated)	Dominated ($4541-dominated)	Dominated
Continual viral load every 6 months	45.6 (42.7–49.0)	19 (−276–320)	34 (25–45)	$292,107 ($17,550-dominated)	Dominated ($27,785-dominated)	Dominated
Continual viral load every 6 months and increased second-line	45.8 (42.8–49.1)	81 (−212–355)	127 (97–160)	$69,140 ($16,409-dominated)	Dominated ($20,685-dominated)	Dominated

Within each treatment initiation stratum, average and incremental cost-effectiveness ratios are calculated based on the total additional cost and QALYs gained.

### Sensitivity analysis

Our sensitivity analysis indicated that the cost of ART, viral load and CD4 cell count testing increased the cost-effectiveness ratios so that increasing second-line use was no longer cost-effective (Supplementary Figure 5). Three parameters, cost and QALY discounting and TDR prevalence, did not change the overall outcome that increasing use of second-line is considered cost-effective when treating at all thresholds for the ranges tested.

Even when the cost of pre-therapy genotyping was reduced by 90%, the scenario of implementing pre-therapy genotyping was still dominated by other strategies at every treatment initiation threshold. Likewise, a 90% reduction in the price of viral load testing alone did not change the cost-effectiveness outcomes of any of the strategies associated to increased viral load testing. This is likely because viral load tests are also used in the baseline scenarios, so the incremental difference in the scenarios with biannual viral loads is limited.

Under the more realistic assumption that second-line treatment is limitedly used, switching all individuals with confirmed virological failure that persist even after adherence counselling is still considered cost-effective when initiating treatment at CD4 <350 (ICER $1437, IQR $643–$3882) and CD4 <500 ($1681, $488–$8491) and very cost-effective when initiating treatment immediately ($563, $433–$792). It should also be noted that when second-line treatment is limitedly used, the prevalence of TDR is predicted to be as high as 30% in 10 years with immediate treatment, highlighting the importance of second-line use (Supplementary Figure 6).

## Discussion

This mathematical model of the Kampala setting predicts that the prevalence of TDR will rise from 6.7% up to between 6.8 and 11.1% over the coming decade. The absolute number of TDR cases is predicted to decline due to the preventative effects of earlier treatment. Among three patient monitoring strategies assessed in this analysis, increasing use of second-line treatment can avert the most TDR infections. Pre-therapy genotyping and twice-yearly viral load monitoring are costly with limited health benefits at a population level, and therefore should not be prioritized in ART programme implementation.

We found that increased use of boosted PI-based second-line treatment is the only cost-effective approach for reducing TDR. Compared to NNRTIs, boosted PIs have a higher genetic barrier (a higher number of mutations are required to overcome drug selective pressure) for the development of drug resistance [31]. Consequently, use of PIs is associated with a lower probability of resistance development during treatment and subsequent transmission of resistance to others [32]. At the JCRC, yearly viral load monitoring is already common practice, as recommended by the WHO [4]. No additional laboratory monitoring is therefore necessary to implement increased use of second-line treatment in this setting.

Increasing viral load testing to more than once per year has a limited impact on TDR prevalence. This is in agreement with data from literature that showed that the risk of virological failure reduces with increased time of virological suppression [33,34]. Combining increased use of second-line treatment with twice-yearly viral load resulted in fewer QALYs gained than increased use of second-line treatment alone. This is due to the fact that increased viral load monitoring will also increase resuppression on first-line therapy [35]. However, the vast majority of resuppressed patients in our dataset went on to fail on first-line therapy again after one year. Therefore, the increased resuppression rate result in two time periods of a patient failing on first-line therapy instead of one time period. Our model assigns slightly lower QALYs to the time individuals spend failing on therapy compared to being successfully suppressed on therapy (see Supplementary Table 2). Therefore, more resuppression on first-line, as with viral load testing every six months, will lead to more instances of viral failure on first-line and therefore slightly lower QALYs on a population level over time. Once individuals are on a boosted PI-based second-line regimen, the likelihood of failure decreases significantly, due to the high genetic barrier [31]. Thus, viral load determination remains of key importance for monitoring ART, but increasing its frequency to twice-yearly does not greatly impact TDR and is not cost-effective.

Pre-therapy genotyping had little added benefit on a population level and is very expensive. Pre-therapy genotyping would potentially have a larger impact on TDR and be most cost-effective if the baseline prevalence of TDR were higher in the modelled scenarios. Indeed, the simulations of pre-therapy genotyping with the lowest ACERs were the simulations in which TDR was the highest (data not shown).

Previous studies have investigated the impact and/or cost-effectiveness of laboratory-based patient monitoring compared to symptom-based patient monitoring [36–40]. The majority have predicted that laboratory-based monitoring was cost-effective or cost-saving, but depends largely on test costs [36–38,39]. One study found that viral load testing every 12 months is more cost-saving than viral load testing every six months, in agreement with our results of viral load testing every six months being cost-ineffective [40]. Just two studies incorporated the preventative effects of laboratory-monitoring techniques on HIV transmission with cost-effectiveness analyses, and found that regular viral load monitoring was highly cost-effective and even cost-saving [36,38]. A study by Phillips *et al*. evaluated virological monitoring while taking into account both drug resistance and HIV transmission [7]. This study concluded that viral load tests every six months would reduce TDR by about 50% compared to clinical monitoring. These results cannot be compared to ours, as our baseline scenario included yearly viral load measurements.

Our mathematical model and cost-effectiveness analysis has several strengths. To our knowledge, our model is the first to include multiple ART intervention strategies into a population-level model that accounts for HIV transmission dynamics, TDR, and cost-effectiveness simultaneously within one dynamic model. Second, our model is also the first to demonstrate the cost-effectiveness of second-line treatment at several treatment initiation thresholds, and the consequences on TDR if second-line has limited availability. Third, this model combines data on transmitted and acquired HIV drug resistance from the same geographic areas and time period, collected within the same research project. Finally, comprehensive cost data were also collected and utilized from the same study site.

This study has some potential limitations. First, data on HIV drug resistance beyond 24 months of ART in resource-limited settings are scarce. While data from high-income countries shows that acquired resistance after two years on therapy diminishes or reaches steady-state [41,42], it could be that acquired resistance after 24 months is as high as 12–24 months acquired resistance rates. If this were the case, it could be that we underestimated future TDR prevalence. Based on our model output, it is unlikely that the outcomes of the different patient monitoring strategies would contradict our results. Second, our predictions rely on the reasonable assumption that drugs used as first-line will remain constant over the coming 10 years, although ART guidelines are subject to change. Third, the cost of second-line is relatively low at the JCRC ($268 per year) compared to tenofovir-based first-line ($223 per year). When the costs of second-line are increased to $466 per year, twice that of a tenofovir-based regimen, increased second-line is no longer considered cost-effective. All other scenarios, however, continue to be dominated by increased second-line. It is therefore of the utmost importance to keep the cost of PI-based second-line drugs as low as possible.

We modelled a setting where second-line is widely used and viral load testing is performed annually. Availability of yearly viral load testing and second-line use is not mirrored across sub-Saharan Africa. We have reported that increased use of second-line is cost-effective when viral load testing is already in place. We cannot say, however, how cost-effective increased second-line use would be in the absence of viral load testing. We attempted to address this issue by modelling a 50–70% reduction in second-line use, and found that that the cost-effectiveness of second-line became stronger or even cost-saving. We could not reliably model the absence of viral load monitoring, as we do not have data to accurately calibrate the model for such an analysis and wanted our model to reflect available data.

## Conclusions

While the prevalence of TDR is predicted to increase with ART initiation at higher CD4 cell count thresholds, the incident cases with TDR are predicted to decrease. Increasing the number of individuals who switch directly to second-line after confirmed first-line failure, in a setting where annual viral load monitoring is already in place, is both cost-effective and reduces TDR at all treatment initiation thresholds. Our observations are particularly relevant in light of the 2013 WHO guidelines which recommend treatment initiation at CD4 <500 cells/µl [4]. With the increasing rollout of first-line treatment, it is imperative to simultaneously expand access to yearly viral load testing coupled with affordable second-line ART, in order to facilitate appropriate switching to second-line ART.

## Supplementary Material

Increasing the use of second-line therapy is a cost-effective approach to prevent the spread of drug-resistant HIV: a mathematical modelling study
