# Supplementary material for: Increasing the use of second-line therapy is a cost-effective approach to prevent the spread of drug-resistant HIV: a mathematical modelling study
Source: J Int AIDS Soc. 2014 Dec 5;17(1):19164. doi: 10.7448/IAS.17.1.19164 (PMC4260459; doi:10.7448/IAS.17.1.19164)
Supplement: Increasing the use of second-line therapy is a cost-effective approach to prevent the spread of drug-resistant HIV: a mathematical modelling study [file JIAS-17-19164-s001.pdf]

Table S1. Variables used to calibrate and accept simulations using the Monte Carlo filtering technique

Parameter used to accept simulations	Values Accepted	Source
Transmitted Drug Resistance (TDR) Prevalence	7.1%-10% in 2009	[1]
Proportion of mutations that make up TDR*	7-27% resistance to protease inhibitors<br>23-43% thymidine analogue mutations (TAMs, encoding for resistance to zidovudine and stavudine)<br>40-60% resistance to non-nucleoside reverse transcriptase inhibitors (NNRTIs)	[1]
HIV Prevalence	7.1%-8.4% between 2005 and 2009	[2,3]
JCRC catchment area population	300,000-372,000 in 2012	Local Data

* The M184V (associated with resistance to lamivudine or emtricitabine) and K65R mutations (associated with tenofovir resistance) were also included in the model, but were not calibrated specifically to the model as these mutations were not observed in the PASER-Surveillance data [1].

Figure S1. Simulations of HIV prevalence 1992-2010; Compared to Ugandan HIV Prevalence Data

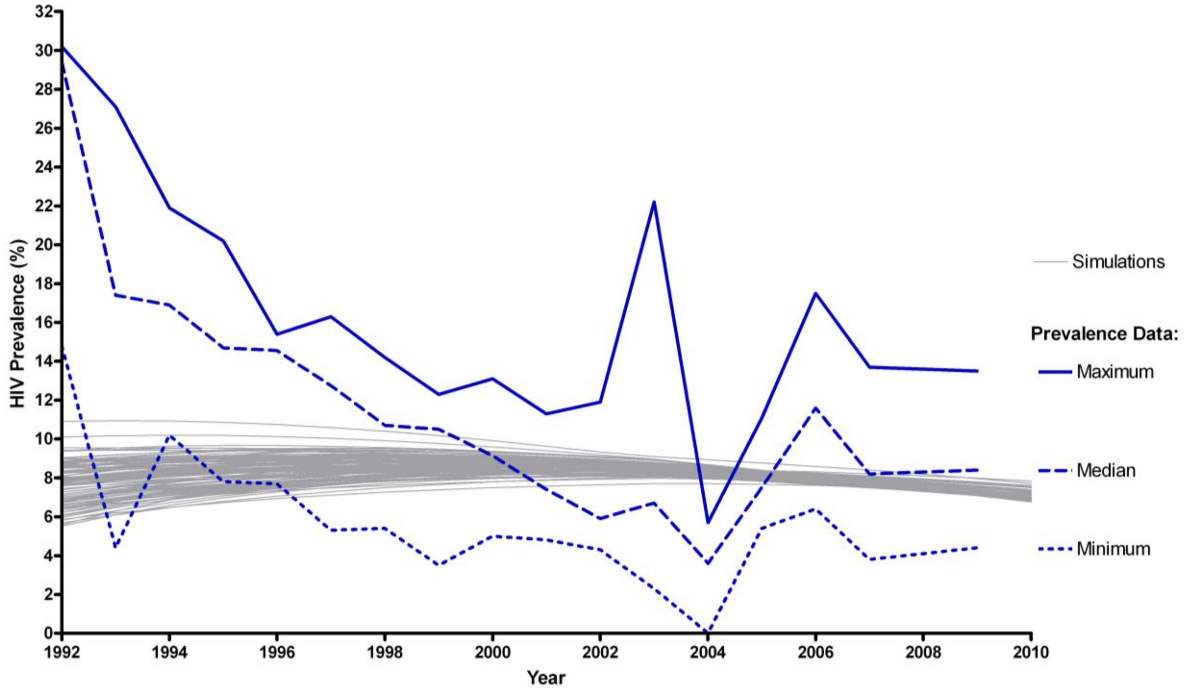

Figure S2. Simulations of HIV population 1992-2012; Joint Research Clinical Centre Catchment Area Population, Kampala, Uganda

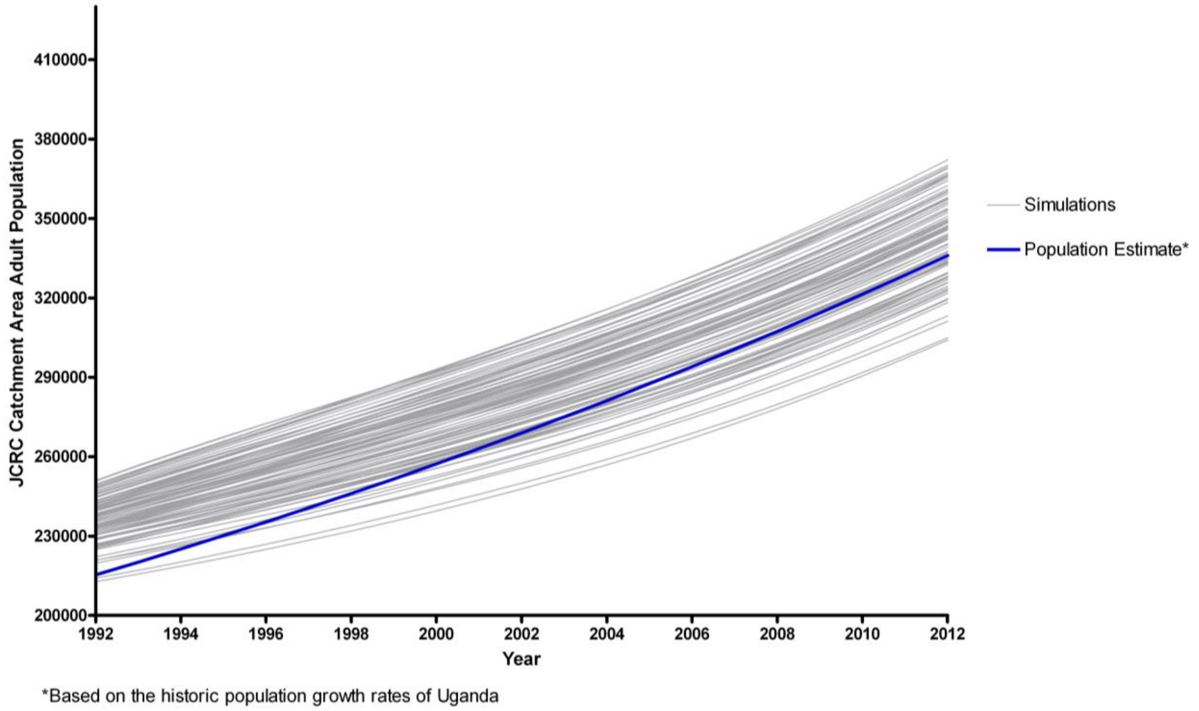

Figure S3. Proportions of CD4 cell count at treatment initiation by treatment initiation guideline

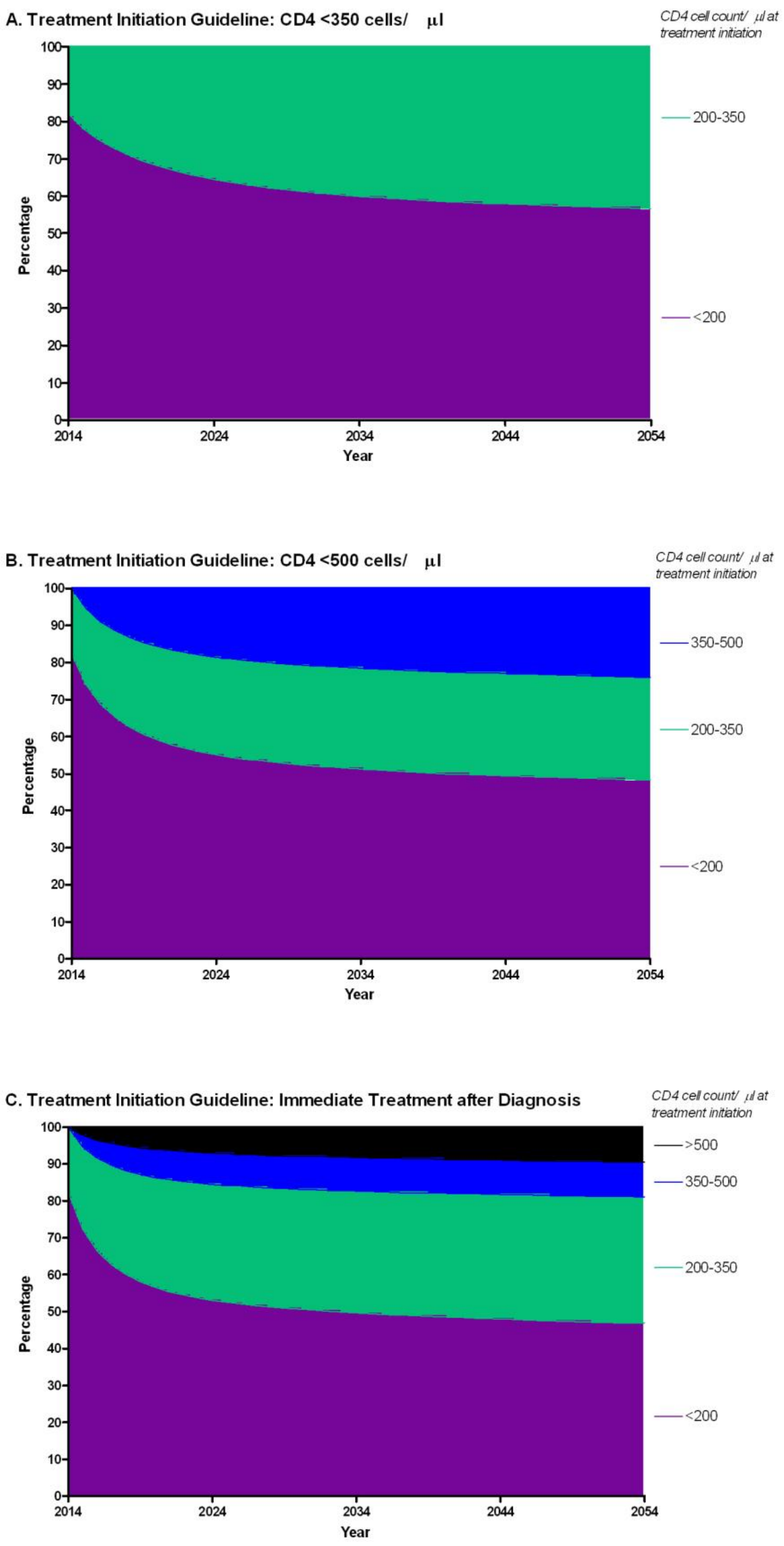

Table S2. Assumed utility weightings for QALYs

Status	Utility Weight*
Susceptible	1.0
Acutely infected	0.94
Chronically infected	0.94
Infected early AIDS stage	0.82
Infected late AIDS stage	0.7
Infected on treatment	0.94
Resistant/failing on treatment	0.82-0.94 (assumption)

*Weights based on a pooled analysis by Tengs and Lin (2002) [4]

Table S3. Costs used in treating opportunistic infections, per unit*

Drug	Unit	Cost, USD
Acyclovir	200mg	$0.20
Azithromycin	500mg	$1.07
Amphotericin B	50mg	$14.00
Ceftriaxone	2g	$4.00
Ciprofloxacin	500mg	$0.20
Fluconazole	200mg	$0.40
RHZ	150/75/400mg	$0.24
RHZE	150/75/400/275mg	$0.48

*All costs collected from the Joint Clinical Research Centre, Kampala, Uganda

Table S4. Costs used in diagnosing opportunistic infections and monitoring HIV, per test

Test/Supply	Cost, USD*
Antigen test	$16.80
Biopsy	$23.60
Complete Blood Count	$5.00
CD4 Test	$14.00
Chest X-ray	$6.00
CSF Analysis	$19.20
Liver function	$26.00
Lumbar puncture	$40.00
Renal function	$20.40
Skin biopsy	$23.60
Stool exam	$12.00
Swab & culture	$12.00
Sputum	$14.40
Urine analysis	$12.00
Viral load test	$55.00

*All costs collected from the Joint Clinical Research Centre, Kampala, Uganda; costs are inclusive of laboratory and hospital personnel (and exclusive cost of outpatient visit)

Table S5. Opportunistic infection rates hospitalization & treatment assumptions*

Opportunistic Infections (OIs)	Percent Hospitalized	Duration of Hospitalization	Drugs used to treat disease:	Additional lab tests needed
Herpes Zoster	0%	N/A	Acyclovir, 5x200mg, 7 days	-
Diarrhea	20%	5 days	Ciprofloxacin, 2x500mg, 5 days	Renal function, stool exam
Tuberculosis	30%	7 days	RHZE 2 months, RHZ 4 months	Chest X-ray, sputum, liver function
Pneumonia	30%	7 days	Ceftriaxone,1x2g, 7 days	-
Oral Candida	0%	N/A	Fluconazole, 1x200mg, 7 days	-
Genital Ulcers	10%	-	Acyclovir, 4x200mg, 14 days	Swab & culture
Esophageal Candida	10%	7 days	Fluconazole, 1x200mg, 7 days	-
Extra Pulmonary TB	50%	10 days	RHZE 2 months, RHZ 10 months	Chest X-ray, sputum, liver function
Cryptococcal Meningitis	100%	17.5 days	Amphotericin B, 1x50mg, 14 days	Lumbar puncture, CSF analysis, antigen test
Kaposi's Sarcoma- Cutaneous	20%	6 days	Start ART	Skin biopsy
Herpes Simplex	10%	7 days	Acyclovir, 4x200mg, 10 days	Swab & culture
Kaposi's Sarcoma- Visceral	100%	7 days	Start ART	Biopsy
Urethritis	40%	7 days	Azithromycin, 1x2g, 7 days	Urine analysis

*Based on expert opinion of one treating physician and head nurse at the Joint Clinical Research Centre in Kampala, Uganda

Figure S4. Yearly transmitted drug resistance prevalence separated out by the following resistance mutation or class: a TAM mutation, M184V mutation, K65R mutation, or resistance to NNRTIs, PIs. Panel A is when treatment is initiated at a CD4 count <350 cells/μl, Panel B at CD4 <500 cells/μl, and Panel C is when treatment is initiated immediately.

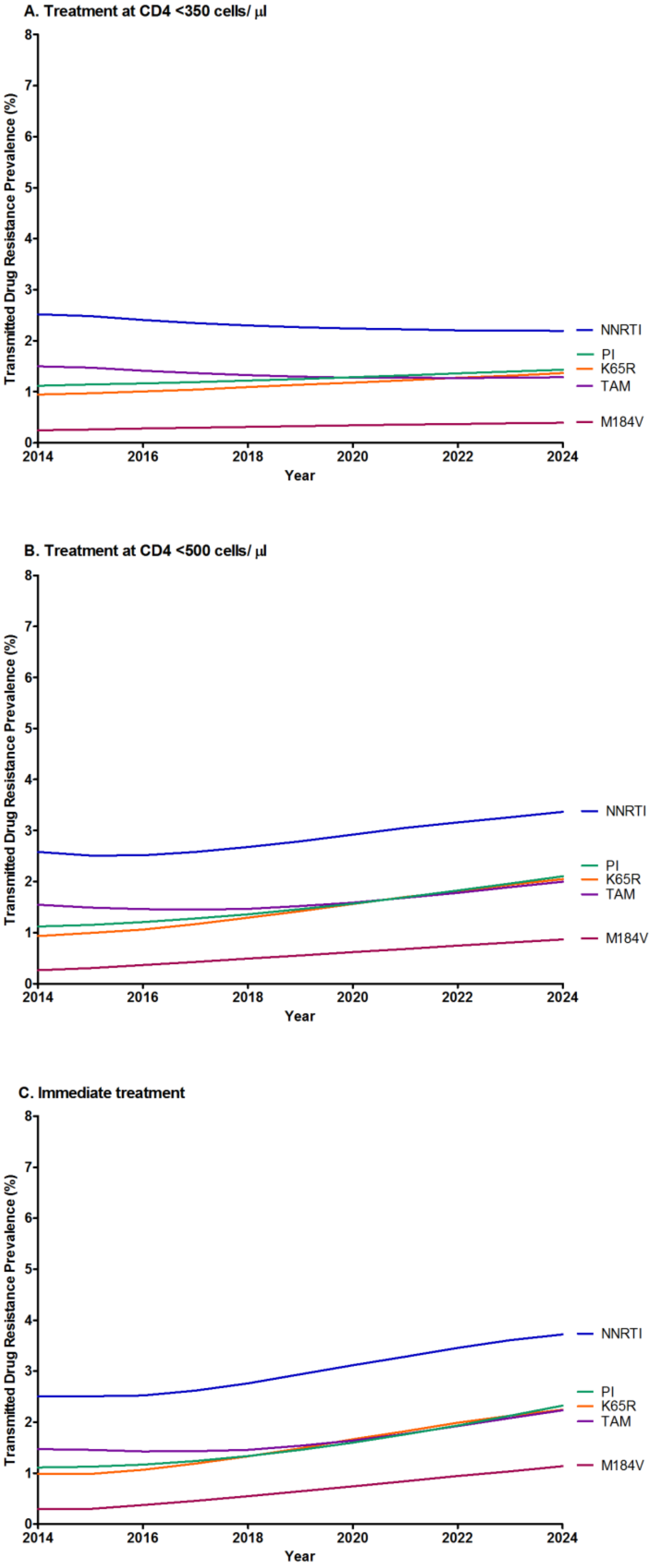

Figure S5. One-way sensitivity analyses of the incremental cost-effectiveness of increasing use of second-line treatment at three different treatment initiation thresholds over 10 years.

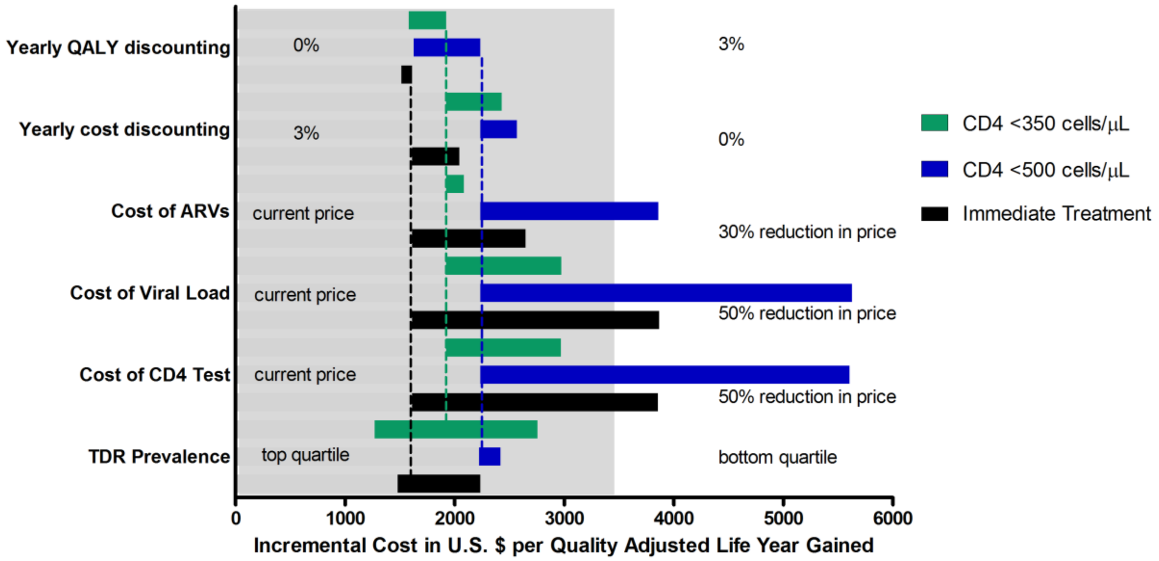

This diagram summarizes the results of a series of one-way sensitivity analyses on the incremental cost-effectiveness of increasing use of second-line treatment at three different treatment initiation thresholds. Each horizontal bar represents the full range of cost-effectiveness ratios produced by varying a given model parameter across its plausible range. The vertical dotted lines represent the incremental cost-effectiveness ratio at each treatment initiation threshold ($1,612 per quality adjusted life year for immediate treatment, $2,234 per quality adjusted life year gained when treating at CD4 <500 cells/μl, and $1,925 per quality adjusted life year gained when treating at CD4 <350 cells/μl). The gray area represents the values that can be considered cost-effective.

Figure S6. Sensitivity analysis: transmitted drug resistance prevalence by treatment initiation threshold when second-line treatment is limitedly available (solid line) versus scaled up to 80-100% (dashed line)

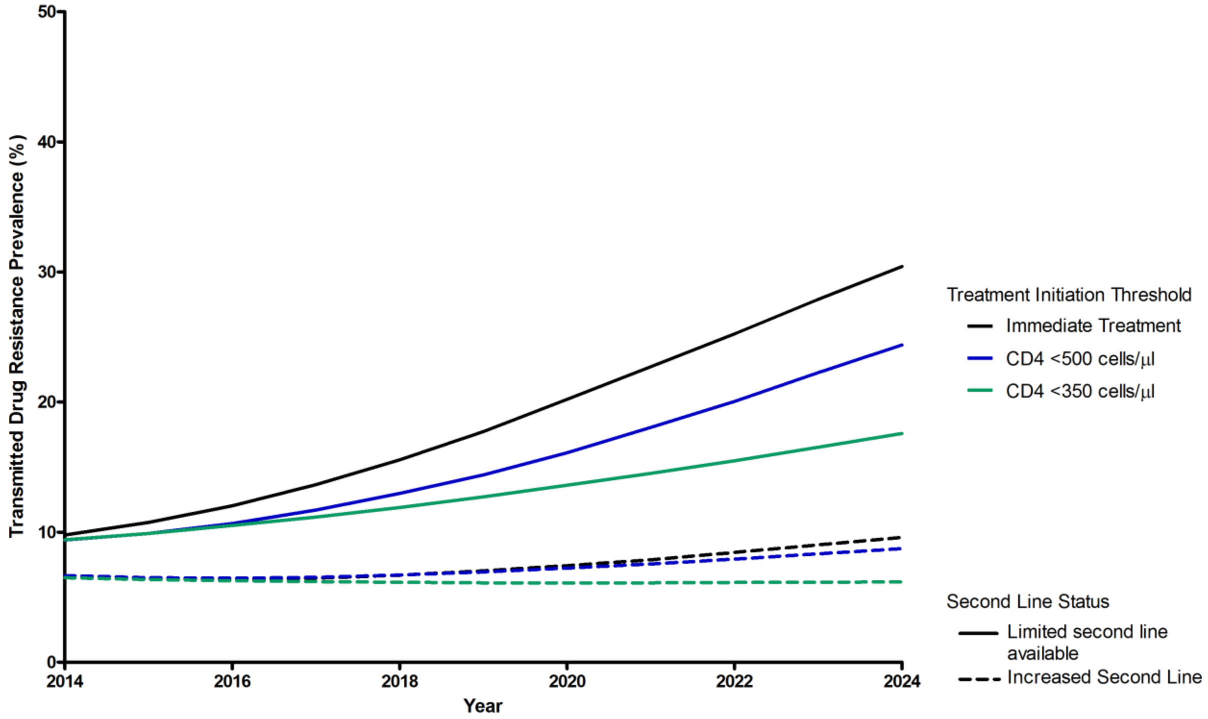

References:

1. Ndembi N, Hamers RL, Sigaloff KC, Lyagoba F, Magambo B, et al. (2011) Transmitted antiretroviral drug resistance among newly HIV-1 diagnosed young individuals in Kampala. AIDS 25: 905-910.
2. Nichols BE, Sigaloff KC, Kityo C, Mandaliya K, Hamers RL, et al. (2014) Averted HIV infections due to expanded antiretroviral treatment eligibility offsets risk of transmitted drug resistance: a modeling study. AIDS 28: 73-83.
3. STD/AIDS Control Programme (2010) The HIV/AIDS Epidemiological Surveillance Report 2010. Kampala: Ministry of Health, Uganda.
4. Tengs TO, Lin TH (2002) A meta-analysis of utility estimates for HIV/AIDS. Med Decis Making 22: 475-481.
